# Uncommon Scurvy Manifestation: Solitary Skin Lesion in an Alcoholic Case

**DOI:** 10.7759/cureus.57652

**Published:** 2024-04-05

**Authors:** Hoda Shabpiray, Saira Khan, Jama Hersi, Mani Khorsand Askari

**Affiliations:** 1 Medicine, University of Toledo, Toledo, USA; 2 Medicine, The University of Toledo Medical Center, Toledo, USA

**Keywords:** high-risk group, alcoholic, solitary skin lesion, uncommon scurvy manifestation, vitamin c deficiency

## Abstract

Scurvy, characterized by vitamin C deficiency, typically manifests with various symptoms, most commonly skin lesions. However, the presentation of a solitary skin lesion is considered atypical. An elderly patient with a history of heavy alcohol consumption presented with a small skin lesion that developed rapidly into a solitary open wound without any preceding trauma. Laboratory analysis revealed severe vitamin C deficiency (<5 μmol/L). The patient showed significant improvement following high-dose vitamin C replacement therapy. This case underscores the potential for scurvy to present with a solitary lower body wound devoid of typical symptoms. It highlights the importance of prompt consideration of vitamin C replacement therapy, particularly in high-risk groups such as alcoholics, by healthcare providers.

## Introduction

Scurvy, also known as vitamin C deficiency, manifests through various clinical signs such as skin lesions (including petechiae, perifollicular hemorrhage, ecchymosis, and coiled hair), joint pain, bleeding into joints and soft tissues, as well as gum problems like gingivitis and hemorrhage, accompanied by delayed wound healing [1]. These symptoms typically emerge after approximately three months of insufficient dietary vitamin C intake [2]. The underlying cause of most symptoms is the disruption of collagen synthesis and metabolism [3]. Over 90% of vitamin C in Western diets comes from fruits and vegetables, and cooking can reduce their vitamin C content by up to 40%. The average American consumes about 75 to 85 mg of vitamin C daily, possibly higher due to vitamin supplements and fortified foods [1]. In the United States, around 7% of adults are estimated to have scurvy, primarily due to factors such as homelessness or alcoholism [1,4]. In this case, we discuss a patient who presented with a solitary open skin lesion, lacking the typical clinical presentations associated with scurvy.

## Case presentation

**Figure 1:**
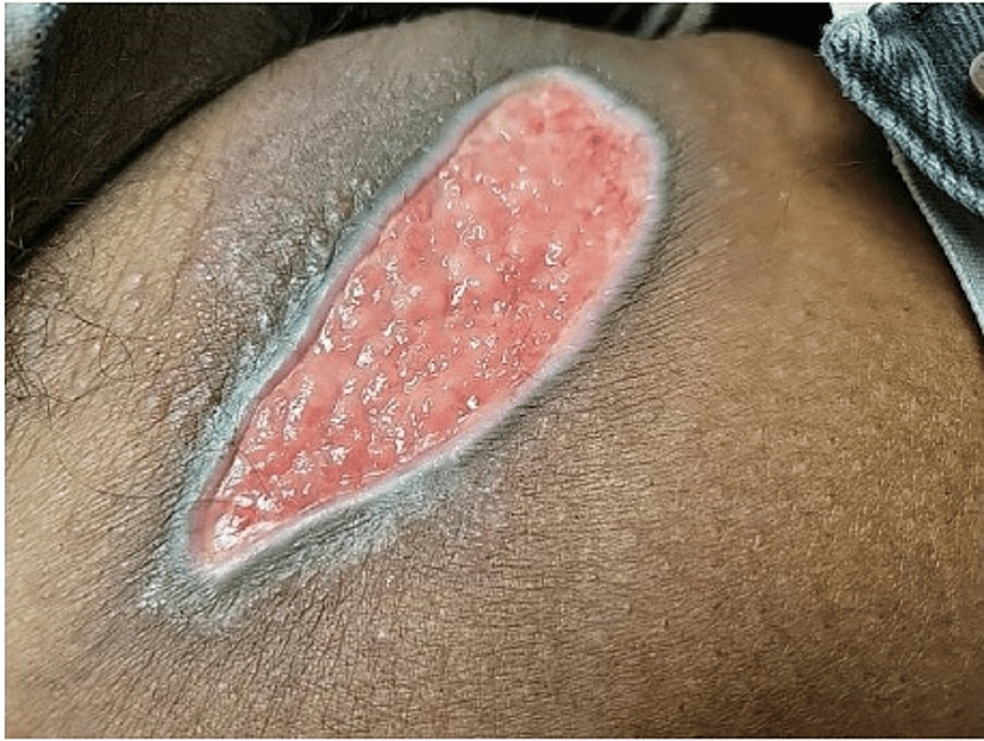
Initial presentation. A 12 cm by 6 cm granulating wound with distinct borders, right thigh inner portion.

An 82-year-old African American male initially presented at the outpatient clinic with a one-week history of a solitary open wound on his right thigh. His medical history included chronic kidney disease (stage 3) and long-term alcoholism, consuming three to four pints of vodka daily for over 20 years. He denied any recent skin injuries or trauma, noting that the wound had started as a small lesion and rapidly progressed over the past few days. He did not report any other skin lesions or additional complaints. On physical examination, a 12 cm by 6 cm granulating wound with distinct borders was observed on the inner part of his right thigh (Figure 1).

Possible diagnoses considered included vasculitis, dermatologic malignancy, especially squamous cell carcinoma (SCC), cutaneous infection like cellulitis or Necrotizing fasciitis (less probable in this case due to lack of fever and septic picture), external tissue damage secondary to trauma, and ulcerative inflammatory conditions, including *Pyoderma gangrenosum*. We discussed with the patient the importance of a skin biopsy to assess for other etiologies. However, he refused the skin biopsy but agreed to laboratory testing. Laboratory tests revealed mild zinc deficiency (56.4 µg/dL, ref range: 60-120 µg/dL), moderate vitamin D deficiency (12.6 ng/mL, ref range: 30-50 ng/mL), and severe vitamin C deficiency (<5 μmol/L, ref range 23 - 114 umol/L). An initial autoimmune work-up showed negative ANCA (antineutrophil cytoplasmic antibody) and normal niacin levels (26 ng/mL ref range 2.5-7.5 ng/mL) (Table 1).

**Table 1:** Patient’s lab values and reference range.

Laboratory test	Patient value	Reference range
Zinc	56.4 µg/dL	60-120 µg/dL
Vitamin D	12.6 ng/mL	30-50 ng/mL
Vitamin C	<5 μmol/L	23-114 μmol/L
Niacin	26 ng/mL	2.5-7.5 ng/mL

**Figure 2:**
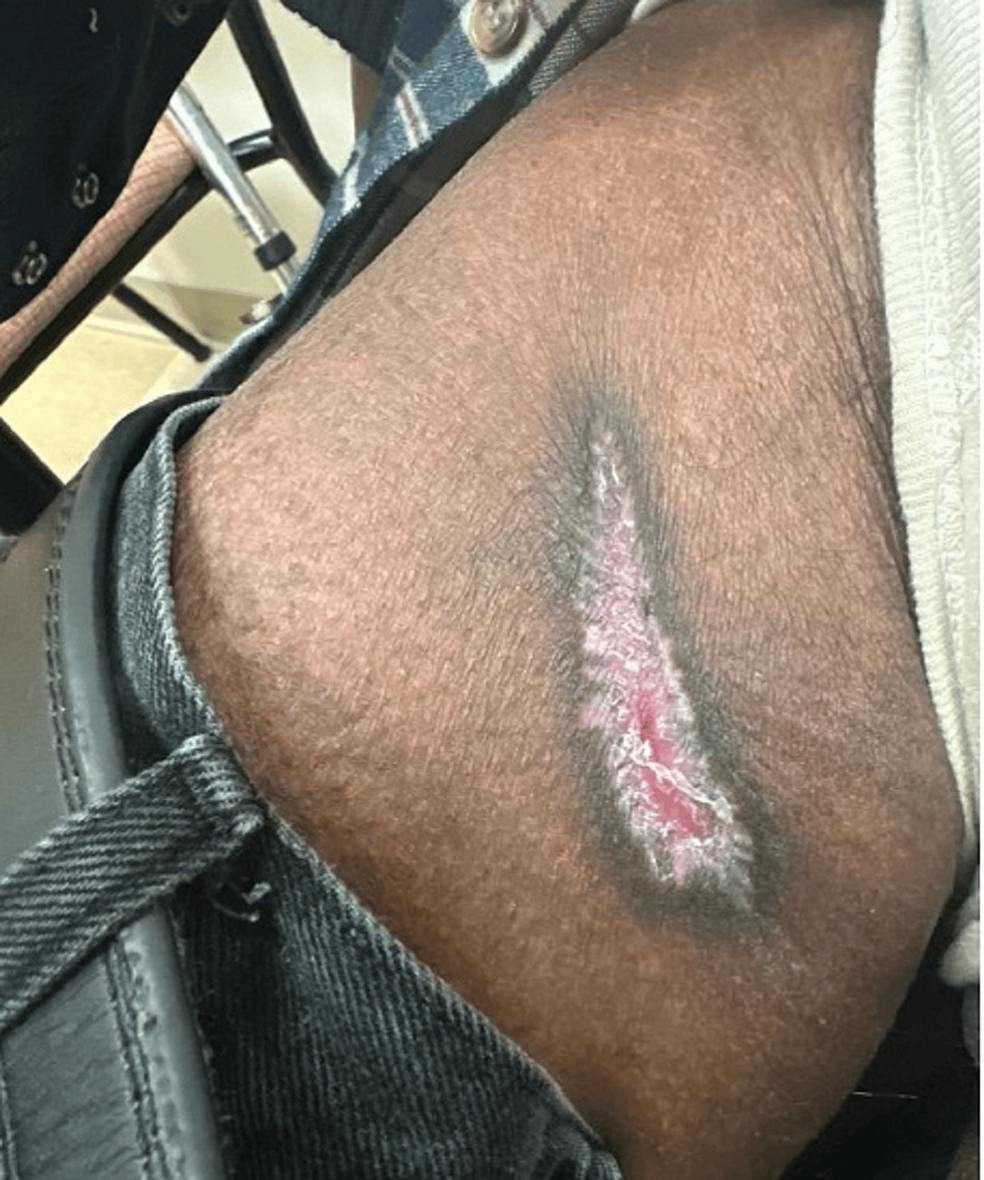
One-month follow-up with replacement therapy. Improvement in the lesion size, 3 cm by 1 cm, with signs of healing.

The patient was counseled on the importance of the intake of fruits and vegetables in the diet and avoidance of alcohol consumption; however, he refused to cut back on drinking but agreed to incorporate more fruits like oranges and lemons in his diet. He was prescribed folic acid 5 mg tablet, thiamin 100 mg tablet daily, and high-dose vitamin C supplementation (1000 mg daily) and scheduled for a follow-up appointment in one month. At the subsequent visit, significant improvement in the lesion size (3 cm by 1 cm) and signs of healing were noted (Figure 2). He was advised to continue vitamin C replacement at 100 mg daily for ten weeks, with a planned repeat follow-up in one month and reassessment of vitamin C levels. 

## Discussion

Identifying scurvy, particularly in unconventional cases like the one discussed, can be challenging in developed nations where clinicians may lack adequate training for its diagnosis. Although scurvy is uncommon in the United States, data comparing its prevalence in Canada show higher rates of vitamin C deficiency at 7.1% versus 2.9%, respectively [5]. This condition tends to affect specific groups in developed countries due to various factors (Table 2) [1,4]. These include individuals living independently with limited access to nutritious food, those with poor dental health unable to consume fruits, individuals avoiding fruits due to dietary constraints, cancer patients experiencing appetite loss, and those with psychiatric disorders like depression, schizophrenia, and anorexia nervosa. Notably, alcoholism is the most common psychiatric condition leading to scurvy [1]. Alcoholics often develop vitamin C deficiency due to multiple reasons, including the absence of vitamin C in alcoholic drinks, inadequate nutrient intake, and reduced absorption of vitamin C in the intestines due to alcohol's effects [6]. Scurvy presents with a range of symptoms such as fatigue, joint pain, and distinct skin signs like bleeding around hair follicles, small red or purple spots, gum inflammation, swollen gums, twisted hair, and slow wound healing [7]. Old scars may soften, and ulcers can form spontaneously or due to minor injuries, especially on the legs [1,8].

**Table 2:** Causes of vitamin C deficiency (scurvy) in industrialized countries

Cause category	Specific causes
Dietary habits	- Inadequate consumption of fresh fruits and vegetables
	- Avoidance of acidic foods due to allergies or gastrointestinal issues
Health and medical conditions	- Underlying conditions like ulcerative colitis, Whipple’s disease, peptic ulcers, and gastroesophageal reflux
	- Poor or absent dentition
Personal preferences	- Dislike for the taste of fruits and vegetables
	- Adherence to bizarre dietary beliefs and food fads
Cancer-related issues	- Decreased nutrient intake due to anorexia or gastrointestinal symptoms from cancer treatment
Psychiatric/behavioral disorders	- Mental health disorders affecting diet, like depression, schizophrenia, or anorexia nervosa

Scurvy diagnosis mainly relies on observing symptoms and a low vitamin C intake history. Initial symptoms of scurvy are characterized by follicular hyperkeratosis and bleeding around hair follicles, which are unique to the condition. Advanced symptoms may manifest as bruising, hair that twists into corkscrew or swan-neck shapes, and gums that are swollen and either red or purple for individuals with teeth. Although direct ascorbic acid measurement isn't standard, blood tests can aid in diagnosis. However, a positive response to vitamin C treatment often confirms scurvy without these tests [1]. Biopsy samples show bleeding around blood vessels and hair follicles, occasionally accompanied by long-term inflammation and iron storage deposits in these areas [1]. The case described, with a single unexplained wound, is rare and noteworthy. It's crucial to understand the diverse ways scurvy can appear, as delayed treatment with vitamin C can lead to severe complications such as seizures, shock, or death [9]. Starting timely and cost-effective treatment and lifestyle changes with appropriate doses of ascorbic acid can lead to swift and complete recovery within a few weeks [10]. The typical treatment involves oral doses of 500 to 1,000 mg daily for two to four weeks or until vitamin C levels are restored [11]. Alternatively, a regimen of 100 mg three times daily can be used, with the duration adjusted based on individual response [1,12]. Maintenance therapy with a low daily dose of 10 mg may also be effective [1]. It is advisable to boost the consumption of fresh produce, mainly citrus fruits and leafy greens, due to their high vitamin C content. Addressing harmful behaviors, such as smoking and heavy drinking, is also crucial [13,14]. 

## Conclusions

The case study exemplifies the diagnostic challenges of scurvy, particularly with unusual symptoms like isolated skin wounds, stressing the need for healthcare providers to consider scurvy in their differential diagnosis, even in well-nourished societies. It highlights the quick symptomatic improvement with vitamin C therapy and stresses the significance of diet and lifestyle adjustments in treatment and prevention. Increased awareness and education about scurvy's diverse manifestations and the pivotal role of nutrition in treatment are essential for healthcare practitioners.
